# Information-entropy enabled identifying topological photonic phase in real space

**DOI:** 10.1007/s12200-024-00113-7

**Published:** 2024-04-29

**Authors:** Rui Ma, Qiuchen Yan, Yihao Luo, Yandong Li, Xingyuan Wang, Cuicui Lu, Xiaoyong Hu, Qihuang Gong

**Affiliations:** 1 State Key Laboratory for Mesoscopic Physics & Department of Physics, Collaborative Innovation Center of Quantum Matter & Frontiers Science Center for Nano-Optoelectronics, Peking University, Beijing, 100871 China; 2 The MOE Key Laboratory of Weak-Light Nonlinear Photonics, TEDA Applied Physics Institute and School of Physics, Nankai University, Tianjin, 300457 China; 3 College of Mathematics and Physics, Beijing University of Chemical Technology, Beijing, 100029 China; 4 Laboratory of Advanced Optoelectronic Quantum Architecture and Measurements of Ministry of Education, Beijing Key Laboratory of Nanophotonics and Ultrafine Optoelectronic Systems, School of Physics, Beijing Institute of Technology, Beijing, 100081 China; 5 Peking University Yangtze Delta Institute of Optoelectronics, Nantong, 226010 China; 6 Collaborative Innovation Center of Extreme Optics, Shanxi University, Taiyuan, 030006 China; 7 Hefei National Laboratory, Hefei, 230088 China; 8 Beijing Academy of Quantum Information Sciences, Beijing, 100193 China

**Keywords:** Information entropy, Kagome model, Topological photonic phase

## Abstract

The topological photonics plays an important role in the fields of fundamental physics and photonic devices. The traditional method of designing topological system is based on the momentum space, which is not a direct and convenient way to grasp the topological properties, especially for the perturbative structures or coupled systems. Here, we propose an interdisciplinary approach to study the topological systems in real space through combining the information entropy and topological photonics. As a proof of concept, the Kagome model has been analyzed with information entropy. We reveal that the bandgap closing does not correspond to the topological edge state disappearing. This method can be used to identify the topological phase conveniently and directly, even the systems with perturbations or couplings. As a promotional validation, Su–Schrieffer–Heeger model and the valley-Hall photonic crystal have also been studied based on the information entropy method. This work provides a method to study topological photonic phase based on information theory, and brings inspiration to analyze the physical properties by taking advantage of interdisciplinarity.

**Graphical Abstract:**

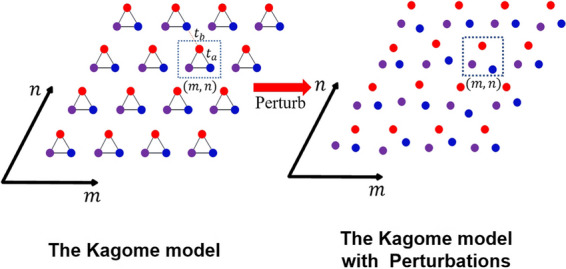

**Supplementary Information:**

The online version contains supplementary material available at 10.1007/s12200-024-00113-7.

## Introduction

The topological photonics plays an important role in the fields of fundamental physics and photonic devices. The Kagome model, the Su–Schrieffer–Heeger (SSH) model and the other topological models are used as a platform to study the novel physics phenomenon [1–5], and guide to design novel photonic devices such as topologically protected laser [6–9] and robust transmission device [10, 11]. Till now, researchers usually judge the topological states in a photonic crystal based on three criterions. The topological invariant, including Chern Number, winding number and *Z*_2_ topological invariant [12–17]; the eigenvalue distributions or gaps in the band of photonics crystal [18–21]; the electric field distributions of the topological states [3, 22]. Almost all the previous methods rely on the band structures in the momentum spaces. However, it is generally complicated to analyze the topological properties in momentum space, especially if there are perturbations in the system. The perturbations will even cause the bandgap closing of topological system, which will bring difficulty to analyze the topological in momentum space.

Here, we propose an interdisciplinary approach to study the topological systems through the information entropy (IE) in real space. Meanwhile, we reveal that the bandgap closing does not correspond to the topological states disappearing. As a proof of concept, the Kagome model is used as an example of theoretical calculation, and the disappearing process of its topological edge states (TESs) is observed with IE. Our method can be used to analyze the TESs mode distributions and topological phase transition. This method can also be extended to SSH model and the valley-Hall photonic crystal. We provide a universal method to study topological photonics and disordered systems based on information entropy theory.

## Results

### Model establishment

**Fig. 1:**
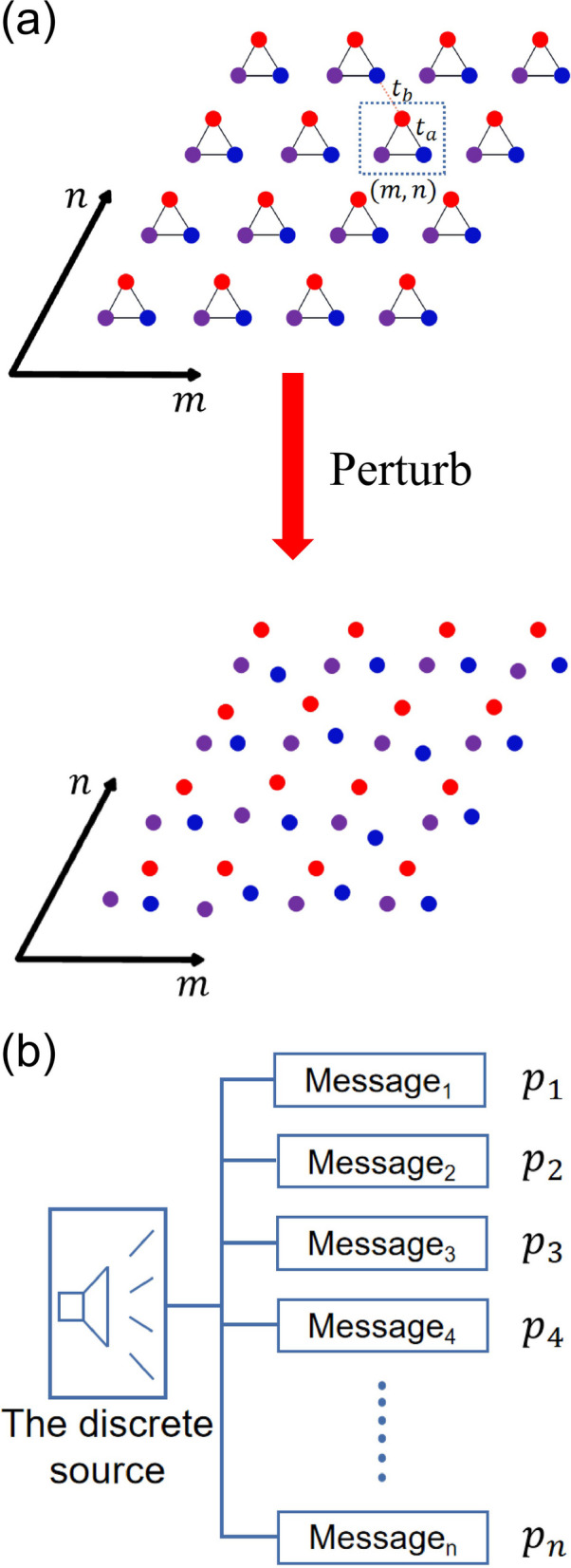
**a** Diagram of the Kagome model and the perturbations causes the system to disorder. **b** Diagram of the discrete sources

Generally, a topological system is a coupled system, like the Kagome model, in which variations in the coupling coefficients significantly impact the emergence of topological states [23, 24]. Figure 1(a) shows the perturbative Kagome model. Assume that there are *M* coupling coefficients in the coupled system by introducing IE, and these *M* coupling coefficients form a set *S*, denoted as $S=\left\{{\kappa }_{1},{\kappa }_{2},\dots ,{\kappa }_{M}\right\}$, where each coupling coefficient is an element of the set, the Hamiltonian of the system thus can be written as

$$\widehat{H}=\sum\nolimits_{i=1}^{M}{\kappa }_{i}\left(\left|B\rangle \langle \left.A\right|\right.+h.c.\right),$$

where *A* and *B* represents arbitrary different atoms, respectively.

It is necessary to establish discrete information sources (ISs) in this set before understanding the IE. The ISs send out random messages, but the number of all possible output messages is finite. The information sent by such an ISs has a certain uncertainty, therefore the concept of IE can be used to describe the ISs. The discrete ISs are shown in Fig. 1(b). All the coupling coefficients in the set *S* are normalized, and the normalization factor is $G=\sum_{i}{\kappa }_{i}$, then all the elements in the set *S* after normalization can be written as $\frac{{\kappa }_{i}}{G}$. Moreover, by taking *N* subsets of the set *S*, each subset can be denoted by ${BIN}_{i}$, for short ${B}_{i}$, where $I=\mathrm{1,2},3,\dots ,N$. Since the magnitude of all coupling coefficients in the set *S* is located in this interval $\left[\left.0,\left(\frac{{\kappa }_{Max}}{G}-\frac{{\kappa }_{Min}}{G}\right)\right]\right.$ after normalization, the interval width of the set *S* can be denoted as ${H}_{S}=\left(\frac{{\kappa }_{Max}}{G}-\frac{{\kappa }_{Min}}{G}\right)$, where ${\kappa }_{Max}$ and ${\kappa }_{Min}$ represents the maximal and minimal elements in the set *S*, respectively.

The elements can be defined in a subset ${B}_{i}$. All the elements in the set *S* that are in the interval $\left[\frac{i-1}{N}\left(\frac{{\kappa }_{Max}}{G}-\frac{{\kappa }_{Min}}{G}\right),\frac{i}{N}\left(\frac{{\kappa }_{Max}}{G}-\frac{{\kappa }_{Min}}{G}\right)\right]$ are placed in the *i* ($i\ne N$) subset ${B}_{i}$. When $i=N$, all elements of the set *S* that are in the interval $\left[\frac{i-1}{N}\left(\frac{{\kappa }_{Max}}{G}-\frac{{\kappa }_{Min}}{G}\right),\frac{i}{N}\left(\frac{{\kappa }_{Max}}{G}-\frac{{\kappa }_{Min}}{G}\right)\right]$ are placed in the *i* ($i=N$) subset ${B}_{i}$. Then all the elements of the set *S* are assigned to this *N* subset ${B}_{i}$, and it is easy to know that the interval width of each subset ${B}_{i}$ is ${H}_{i}=\left(\frac{i}{N}-\frac{i-1}{N}\right)\left(\frac{{\kappa }_{Max}}{G}-\frac{{\kappa }_{Min}}{G}\right)=\frac{1}{N}\left(\frac{{\kappa }_{Max}}{G}-\frac{{\kappa }_{Min}}{G}\right)$. Any coupling coefficient ${\kappa }_{i}$ in the set *S* is random in a coupling system, but all possible coupling coefficient ${\kappa }_{i}$ do not exceed the range determined by the *N* subsets ${B}_{i}$ above. In other words, all the possible coupling coefficients ${\kappa }_{i}$ are finite under the range constraints of the *N* subsets ${B}_{i}$ above. Then the discrete ISs are established. The coupling terms of the Hamiltonian of the system are rearranged in these subsets ${B}_{i}$, and the arrangement results are shown as follows:

$$\begin{array}{ccc}\widehat{H}& =& G\sum\limits_{i=1}^{M}\frac{{\kappa }_{i}}{G}\left(\left|B\rangle \langle \left.A\right|\right.+h.c.\right)\\ & =& G\sum_{i=1}^{N}\sum_{j=1}^{{\text{card}}({B}_{i})}\frac{{\kappa }_{j}}{G}\left(\left|B\rangle \langle \left.A\right|\right.+h.c.\right).\end{array}$$

By establishing *N* subsets ${B}_{i}$ in the set *S*, the discrete source can be established. The normalizing factor term $G$ in the above equation is a constant when the coupling system is determined, and term $\sum_{j=1}^{{\text{card}}({B}_{i})}\frac{{\kappa }_{j}}{G}\left(\left|B\rangle \langle \left.A\right|\right.+h.c.\right)$ describes the contribution of each subset ${B}_{i}$ to the whole coupling system. The probability of each subset ${B}_{i}$ can be defined as ${p}_{i}=\frac{{\text{card}}\left({B}_{i}\right)}{{\text{card}}\left(S\right)}$, and the probability of each subset ${p}_{i}=\frac{{\text{card}}\left({B}_{i}\right)}{{\text{card}}\left(S\right)}$ is the probability ${p}_{i}$ in the IE expression $\sigma =-\sum_{i}{p}_{i}{\text{log}}{p}_{i}$ [25–28].

Through this method, the IE of different coupling structures can be solved, and the IE obtained in this way is referred to as the physical entropy (PE). Table 1 shows the 50 random coefficients and statistical results of those coefficients. According to the probability of each subset obtained by statistics, the IE of the coupling system can be calculated.

**Table 1:** Statistics for coupling coefficients in a coupled system with a random distribution of coupling coefficients

*BIN* interval	Coupling coefficients located in different *BINs*	Probability of *BINs*
0–1	0.8, 0.5, 0.0, 0.8, 0.2, 0.8, 0.2	14%
1–2	1.5, 1.3, 1.4, 1.5, 1.9	10%
2–3	2.6, 2.4, 2.6, 2.9	8%
3–4	3.6, 3.7, 3.9, 3.0, 3.2, 3.1, 3.9	14%
4–5	4.8, 4.2, 4.7, 4.2, 4.3	10%
5–6	5.3, 5.0, 5.0, 5.7, 5.6, 5.6, 5.5, 5.8	16%
6–7	6.9, 6.4, 6.2, 6.1	8%
7–8	7.7	2%
8–9	8.8, 8.2, 8.1, 8.8, 8.0, 8.4	12%
9–10	9.5, 9.7, 9.1	6%

### Physical entropy

**Fig. 2:**
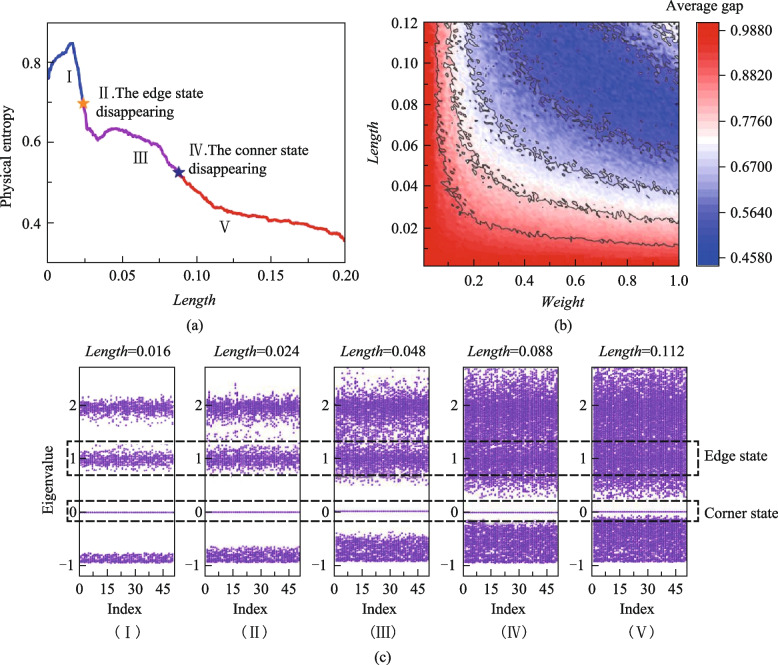
**a** Variation of PE with *Length* of the Kagome model, and the point where the edge state and corner state disappearing correspond to the point where IE decreases swiftly. For Kagome model, the edge state and the corner state have different points where the TESs disappearing. The bandgaps are shown in c(II) and c(III). **b** Diagram of the phase transition of the Kagome model. The red area represents the topological phase and the blue area represents the trivial phase. **c** Band gap state of the Kagome model with different perturbation scale *Length*

In Kagome model (the SSH and the valley-Hall model is also analyzed in the Supplementary Materials (SM) III), when some small perturbations are applied to the topological system, the coupling relationship will change but the mode distributions can change slightly because of its robustness. However, if these perturbations are large enough, the TESs will be transformed into bulk states, then the topological phase transition occurs in the system. To describe the perturbation size of the system, we define two physical quantities as the perturbation scale (denoted as *Length*, representing the ratio of the position offset of a lattice to the lattice constant) and the perturbation rate (denoted as *Weight*, representing the ratio of the number of deviated lattices to the total number of lattices in the system). Assuming that the position offset of all lattices caused by perturbations cannot exceed the lattice constant, the value range of *Length* is $\left[\left.\mathrm{0,1}\right]\right.$, and the range of *Weight* is $\left.\left[\mathrm{0,1}\right.\right)$. The *Weight* is determined before perturbations occur, and the lattices are shifted to random directions in a certain amount according to the size of the *Length.* We show that the size of the *Length* directly affects the bandgap state of the Kagome system, as shown in Fig. 2(c).

By calculating the influences of various disturbances on the Kagome model, the disturbance that applied randomly has a certain probability of closing the band gap of the topological system. For each set of determined *Length* and *Weight*, there can be an infinite number of possible perturbations in coupling system. Here, the eigenvalue distributions of 50 Kagome systems are analyzed after adding different random perturbations with the same *Length* and *Weight*. Due to the different lattices randomly selecting for each disturbance, the band structures of each disturbance will also be different. However, by taking these 50 systems as a statistical global system, no matter how the random perturbations are applied, the system as a global system reflects the characteristics of band gap closing when the *Length* of Kagome system is greater than a certain value.

The statistical results show that when the *Length* is greater than 0.024, the band gap of Kagome system tend to close, meaning that the disappearing of TESs. However, the corner states still remain. This phenomenon can be reflected in the IE characteristics of the coupled system. Figure 2(a) shows that the PE will decrease quickly at *Length* = 0.024, meaning that the coupling system have a huge change. Moreover, the corner-state gap of Kagome model will tend to close in the case of *Length* > 0.08. The PE decreases again at *Length* = 0.088. For other topological systems, the details on the phenomenon of band gap closing caused by perturbations can refer to SM II, including the SSH system and the valley-Hall photonics crystal.

Since the PE is a statistical analysis of the coupling parameters, some small perturbations will cause new coupling parameters in the system. Since the *Length* changes from 0 to a small value at the beginning, this process will inevitably lead to the generation of new coupling parameters, and the PE increases at the moment when the system begins to be disturbed. However, when the *Length* continues to change there are no new coupling parameter generated. The PE tends to be a stable value. Furthermore, this phenomenon can be illustrated by analyzing the PE change of the system, the total PE change of the system $\Delta \sigma$ is as follows:

$${\Delta \sigma =\sum_{i=k}^{N}{p}_{i}{\text{log}}{p}_{i}|_{\text{new}}}-{\sum_{i=k}^{N}{p}_{i}{\text{log}}{p}_{i}|_{\text{old}}}.$$

The change of IE of the system can be measured by the ratio of IE change $\Delta \sigma$ to ${\sum_{i=k}^{N}{p}_{i}{\text{log}}{p}_{i}|}_{{\text{old}}}$. The ratio is defined as $\eta =\left|\frac{\Delta \sigma }{-\sum_{i}^{N}{p}_{i}{\text{log}}{p}_{i}}\right|$. It can be proved that the ratio satisfies the following characteristics (the detailed certification process is provided in SM II).

- 1) If the magnitude of the coupling coefficient change caused by *Length* is less than the width *H* of *BIN*;
- 2) If the magnitude of the coupling coefficient change caused by *Length* is close to or equal to the width *H* of *BIN*;
- 3) If the magnitude of the coupling coefficient change caused by *Length* is much larger than the width *H* of *BIN*, but the coupling coefficient changes $\Delta \kappa \left(Length\right)$ caused by the perturbation scale *Length* is not greater than the normalized factor $G$.

In these three cases above, the ratio $\eta =\left|\frac{\Delta \sigma }{-\sum_{i}^{N}{p}_{i}{\text{log}}{p}_{i}}\right|$ is a small quantity. The calculated result of PE is robust. The perturbations hardly change the IE value, proving why IE almost retains a constant value in the initial state when adding perturbations. Meanwhile, the energy gap of the system is gradually reduced but not closed in the band gap, which also reflects the robustness of the system. When *Length* = 0.024, the coupling distribution between lattices has a qualitative change. The state of the main coupling coefficients in the original coupling system is broken, a large number of new disordered coupling coefficients appear. Some lattices have a strong interaction due to the close distance. Since the coupling parameters will be normalized by the factor $G$ when constructing the discrete IS *Bin*, the appearance of the strong interaction directly affects the normalization factor of PE, and the original coupling distribution is completely destroyed. The IE will decrease at a very fast rate at that time. The sudden change of the coupling distribution reflects the closing of the energy gap, which means the topological phase transition occurs and disappearing of TESs. The detailed deduction process is provided in SM II. The phase diagram shown in Fig. 2(b) also demonstrates the phase transition process of Kagome model with the disturbance increasing. The average gap is used to describe the closing state of the band gap. The decreasing trend of the average gap indicates the gradual closing process of the band gap. The IE can be used as a new criterion of TESs, and to evaluate the robustness of a TES in topological systems. When the disturbance of the system is not large, the PE almost retains a constant value in the initial state of the perturbation. Further, the application of IE method in complex lattice is discussed in SM IV. The IE method can still work in the complex lattice, such as the super-SSH model [29]. A discussion about the case that the disturbance is applied on the potential of lattice site in the Kagome model is also provided in SM IV as the generalization of the IE method.

### Field entropy

**Fig. 3:**
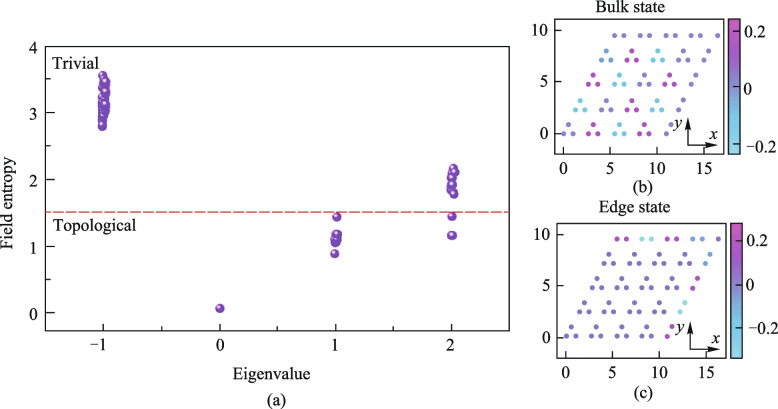
**a** FE of the Kagome model. The electric field distribution of the TES are the states with a low FE, and the bulk states is a series of states with a high entropy of FE. **b** Bulk state of the Kagome model; **c** TES of the Kagome model

In addition, IE can be further extended. In addition to the coupling state in coupling system, the mode distributions can also be statistically analyzed based on IE method. The mode distributions are also necessary to demonstrate the propagation of TESs. Similar discrete sources are defined according to the definition of PE. Taking the pixel matrix of the mode distribution diagram, in which there are *M* mode values, and these *M* coefficients form a set *S*, denoted as $S=\left\{{E}_{1},{E}_{2},\dots ,{E}_{M}\right\}$. The electric field can be normalized by similar methods, and the subsets can be divided as well. Each subset ${B}_{i}$ is a statistical unit that completes statistics with the IE. The electric field intensity is treated in the same discrete unit as the same class, and only distinguish the field intensity differences between different *BINs*. In addition, we further discretized the field intensity distribution into small pixel cells on a plane, and then represented the intensity in the discrete pixel grid as the average of the field intensity in the pixel grid. Thus, the construction of discrete IS is realized in an electric field intensity distribution. Then, the probability of each subset ${B}_{i}$ can also be defined as ${p}_{i}=\frac{{\text{card}}\left({B}_{i}\right)}{{\text{card}}\left(S\right)}$, and the IE of the electric field distribution can then be solved, too. The field entropy (FE) of the unperturbed system can be obtained, shown in Fig. 3.

**Fig. 4:**
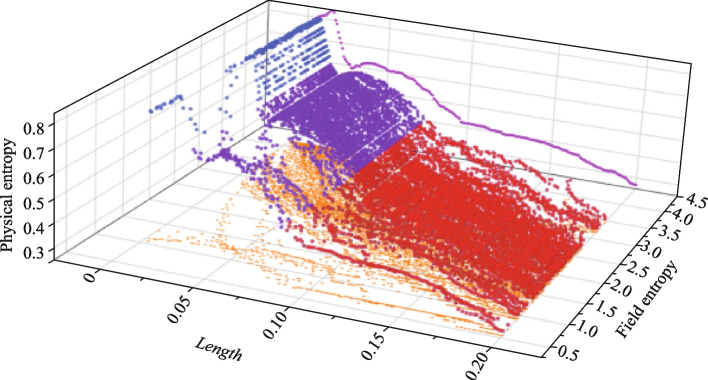
Relationship between the PE and FE of the Kagome model. The blue, purple and red points are the relationship between the PE and FE with different perturbation scales *Length,* and those points combines a curved surface. The yellow points are the projector of the surface, meaning that the FE of the Kagome model with the perturbation scale growing. The process of the FE gap closing can be observed. The pink points are variation of PE with *Length* of the Kagome model

In a topological system, the mode distributions of TESs are only localized on the boundary and its position information is determined, while the bulk state mode distributions are dispersed. Due to the irregular distributions of bulk states, the IE value of the bulk states is higher than that of the TESs. Furthermore, the relationship between the FE and PE is analyzed and solved. The results show that FE can also be used as a phase transition characterization of topological systems, seen in Fig. 4.

As the disturbances increase, the PE will drop dramatically at the position of *Length* = 0.02, which indicates the bandgap closing and topological phase transition occurring. Meanwhile, the difference between the high entropy state and the low entropy state of FE will also decrease. The FE gap can be defined as the difference between the high entropy state and the low entropy state of FE. It is easy to find that the FE gap closes after the position where the PE phase transition occurs. This shows that the FE gap can be used as a physical quantity to describe the phase transition of TESs. Figure 4 also reveals that the bandgap closing does not correspond to the TESs disappearing because the point where the PE sharply decreases does not correspond to the point where the difference in FE disappears. The concept of IE can describe both the change of electric field and the change of coupling structure of topological system. The IE is a good platform for describing topological phase transitions and TESs.

## Conclusion

We propose a method to study the topological systems in real space by analyzing the IE, and reveal that the bandgap closing does not correspond to the TESs disappearing. The PE can reveal the topological phase transition by describing the coupling state change of the system without setting up an experimental platform for measurement; meanwhile the FE can distinguish the TESs from the bulk states through the differences of IE, which can also be used to describe whether the electric fields excited in the micro-nano structure of the photonic crystal are TESs. Thus, the topological photonic phase can be identified by both the PE and FE. The IE has great application potential in the experimental realization of real photonic crystal systems. The IE method is an effective method to analyzing the disordered topological systems for multiple topological models.

### Supplementary Information

**Supplementary Material 1. **

## Data Availability

The data that support the findings of this study are available from the corresponding author, upon reasonable request.
