# Supplementary material for: Information-entropy enabled identifying topological photonic phase in real space
Source: Front Optoelectron. 2024 Apr 29;17(1):11. doi: 10.1007/s12200-024-00113-7 (PMC11056353; doi:10.1007/s12200-024-00113-7)
Supplement: Supplementary file 1 — Supplementary Material 1. [file 12200_2024_113_MOESM1_ESM.pdf]

Supplemental Material

Information-entropy enabled identifying topological photonic phase in real space

Rui Ma,[1,*] Qiuchen Yan,[1,*,†] Yihao Luo,[2] Yandong Li,[1] Xingyuan Wang,[3] Cuicui Lu,[4,‡] Xiaoyong Hu[1,5,6,7,§] and Qihuang Gong[1,5,6,7]

[1]State Key Laboratory for Mesoscopic Physics & Department of Physics, Collaborative Innovation Center of Quantum Matter & Frontiers Science Center for Nano-optoelectronics, Beijing Academy of Quantum Information Sciences, Peking University, Beijing 100871, P. R. China

[2]The MOE Key Laboratory of Weak-Light Nonlinear Photonics, TEDA Applied Physics Institute and School of Physics, Nankai University, Tianjin 300457, China

[3]College of Mathematics and Physics, Beijing University of Chemical Technology, Beijing 100029, China.

[4]Laboratory of Advanced Optoelectronic Quantum Architecture and Measurements of Ministry of Education, Beijing Key Laboratory of Nanophotonics and Ultrane Optoelectronic Systems, School of Physics, Beijing Institute of Technology, Beijing 100081, P. R. China

[5]Peking University Yangtze Delta Institute of Optoelectronics, Nantong, Jiangsu 226010, P. R. China

[6]Collaborative Innovation Center of Extreme Optics, Shanxi University, Taiyuan, Shanxi 030006, P. R. China

[7]Hefei National Laboratory, Hefei 230088,China

** The authors contributed equally to this work.*

Corresponding Author: qiuchenyan@pku.edu.cn, cuicuilu@bit.edu.cn, xiaoyonghu@pku.edu.cn

Contents

I. The concept of the information entropy and the advantage of the information entropy

II. The theory of the information entropy analyzing the coupling system

III. The extension of information entropy method in other topological systems, including Su-Schrieffer-Heeger (SSH) model and the valley-Hall photonic crystal.

IV. The discussion about the information entropy method can be generalized to other complex lattice models and the potential of lattice site in coupling system.

I. The concept of the information entropy and the advantage of the information entropy

The information entropy was provided by Claude Shannon in 1948[25]. And the mathematical form is the following:

$$\sigma = -\sum_{i}^{N} p_i \, log \, p_i$$

The formula above is similar to the expectation. Therefore, the information entropy can be interpreted as the expected value of the random variable $\frac{1}{log\, p(x)}$, where x is drawn according to probability mass function $p(x)$. In the other words, the mathematical formula$\frac{1}{log\, p(x)}$is the measurement standard of the information, and the logarithmic form is the only mathematical form to describe the amount of information.

Here, three mathematical properties will be provided below:

1. Normalization: For a binary discrete system, the entropy of the system must satisfy the following:

$$H_2\left(\frac{1}{2}, \frac{1}{2}\right) = 1$$

2.Continuity: For a binary discrete systems, the entropy of the system $H_2(p, 1-p) = 1$ must be a continuous function of the independent variable p.

3.Grouping: the entropy of the system must follow the combination rule:

$$H_m(p_1, p_2, \dots, p_m) = H_m(p_1 + p_2, p_3, \dots, p_m) + (p_1 + p_2) H_2\left(\frac{p_1}{p_1 + p_2}, \frac{p_2}{p_1 + p_2}\right)$$

So, it can be proved that H_m must be of the form:

$$H_m(p_1, p_2, \dots, p_m) = -\sum_{i}^{N} p_i \, log \, p_i$$

The normalization and the continuity is easy to demonstrate, and if the function H_m satisfies the Grouping, any polycomponent system can be transformed to a binary discrete systems with the grouping.

In fact, Shannon also provided the advantages about the logarithmic form of the information entropy [25].

II. The theory of the information entropy analyzing the coupling system.

In the text, we define the probability of each subset $p_i = \frac{card(B_i)}{card(S)}$, then the information entropy was introduced into the topological system. The mathematical definition of physical entropy is given in the article. Here, more details on the phenomenon of band gap closing caused by perturbations were provided, and the influence of perturbation on physical entropy is discussed below:

If perturbation is applied to the system, assuming that the perturbation rate W is a small amount (e.g. $W \leq 5\%$), then the perturbation scale L was changed. There are only two coupling parameters that need to be changed. The two disturbed coupling parameters can be randomly selected. Now we're going to talk about it in four different cases:

1. If the magnitude of the perturbation scale L is less than the interval width H_i of the subset B_i, Such a change does not result in a change in the probability p_i of a subset B_i, in which case the information entropy σ will not change. The mathematical proof is as follows:

According to the previous assumption, if the perturbation rate W is a small quantity,

then the total coupling coefficient of perturbation is $W \times M \ll M$. If the magnitude of perturbation scale L is less than the interval width of the subset, it can be considered that $\kappa_i \pm \Delta\kappa(L) \approx \kappa_i$ at this time. Each coupling coefficient κ_i almost does not change, and the distribution of all elements in the set S in this N subset does not change. The probability of each subset p_i does not change, so the information entropy $\sigma = -\sum_i^N p_i \log p_i$ does not change.

2. If the magnitude of the perturbation scale L is close to or equal to the interval width H_i of the subset B_i. At this time, the disturbance will cause the probability p_i of the subset B_i to change, and the information entropy will change at this time. The following is a detailed discussion:

According to the previous assumption, if the perturbation rate is a small quantity W, then the total coupling coefficient of perturbation is $W \times M \ll M$. For the convenience of differentiation, the coupling coefficient κ_i of disturbance is re-denoted as $\kappa_{j,k}$, where $j = 1,2,3,\cdots,W \times M$. It means that this is the *j-th* coupling coefficient of disturbance. $k = 1,2,3,\cdots,N$, and the k means the subset to which the coupling coefficient $\kappa_{j,k}$ of the disturbance belongs before the disturbance is $B_{j,k}$. If the magnitude of the perturbation scale L is close to or equal to the interval width H_i of the subset B_i, it can be considered that the coupling coefficient $\kappa_{j,k} \pm \Delta\kappa(L)$ of the perturbation in the subset $B_{j,k}$ after the perturbation does not belong to the original subset $B_{j,k}$, but belongs to the new subset $B_{j,k \pm Floor[\Delta\kappa(N \times L)] \pm 0 or 1}$ (because the interval width of each subset B_i is $H_i = \frac{1}{N}$, so it is necessary to round $\frac{L}{1/N}$ down, then the interval crossing degree caused by L should be found. The item $\pm 0 \text{or} 1$ was caused

by the remainder of the integer). Since the change is random, $k \pm Floor[\Delta\kappa(N \times L)] \pm 0or1$ is a constraint on the possible subset after the change. Since the coupling coefficient $\kappa_{j,k}$ of each change changes from the original subset $B_{j,k}$ to the new subset, for simplicity, the new subset is denoted as $B_{j,ne}$, $ne = k \pm Floor[\Delta\kappa(N \times L)] \pm 0or1$.

Since $W \times M$ coupling coefficients changed, the entropy change caused by these coupling coefficient changes can be calculated as follows:

Before the change:

$$\sigma_{old} = -\sum_{i}^{N} p_i \log p_i = -\sum_{i \neq k,ne}^{N} p_i \log p_i \,|_{old} - \sum_{i=k,ne}^{N} p_i \log p_i \,|_{old}$$

After the change:

$$\sigma_{new} = -\sum_{i}^{N} p_i \log p_i = -\sum_{i \neq k,ne}^{N} p_i \log p_i \,|_{new} - \sum_{i=k,ne}^{N} p_i \log p_i \,|_{new}$$

At that time, it is known that the elements in all subsets B_i do not change, and the probability p_i corresponding to the subset B_i does not change. So, the relationship is as follows:

$$\sum_{i \neq k,ne}^{N} p_i \log p_i \,|_{old} = \sum_{i \neq k,ne}^{N} p_i \log p_i \,|_{new}$$

Therefore, the change in entropy is mainly provided by the difference between $\sum_{i=k,ne}^{N} p_i \log p_i \,|_{old}$ and $\sum_{i=k,ne}^{N} p_i \log p_i \,|_{new}$. In the following, we will mainly analyze the differences between $\sum_{i=k,ne}^{N} p_i \log p_i \,|_{old}$ and $\sum_{i=k,ne}^{N} p_i \log p_i \,|_{new}$.

It is easy to know that the change of the $W \times M$ coupling coefficients is concentrated in these two terms, and $W \times M \ll M$, then assuming that each subset B_i has a change in the d_i elements, and $d_i < W \times M$. For simplicity, assuming that *A* subsets lose an element, and *C* subsets gain an element, so every set that loses elements loses d_u elements, and every set that gains elements gains d_v elements, where $u = 1,2,3,\cdots,A$, $v = 1,2,3,\cdots,C$. And since the behavior of coupling coefficient across subsets is discussed at this time, we replace all corner scripts *i* with the previous corner scripts *j*,*k*, then:

Before the change:

$$\sum_{i=k,ne}^{N} p_i \, log \, p_i \,|_{old} = \sum_{k=1}^{A} p_{j,k} \, log \, p_{j,k} + \sum_{k=1}^{C} p_{j,k} \, log \, p_{j,k}$$
$$= \sum_{k=1}^{A} \frac{card(B_{j,k})}{card(S)} log \frac{card(B_{j,k})}{card(S)}$$
$$+ \sum_{k=1}^{C} \frac{card(B_{j,k})}{card(S)} log \frac{card(B_{j,k})}{card(S)}$$

After the change:

$$\sum_{i=k,ne}^{N} p_i \, log \, p_i \,|_{new} = \sum_{k=1}^{A} p_{j,k} \, log \, p_{j,k} + \sum_{k=1}^{C} p_{j,k} \, log \, p_{j,k} \,|$$
$$= \sum_{k=1}^{A} \frac{card(B_{j,k}) - d_u}{card(S)} log \frac{card(B_{j,k}) - d_u}{card(S)}$$
$$+ \sum_{k=1}^{C} \frac{card(B_{j,k}) + d_v}{card(S)} log \frac{card(B_{j,k}) + d_v}{card(S)}$$

The difference between the above two formulas is obtained:

$$
\begin{aligned}
\Delta\sigma &= \sum_{i=k,ne}^{N} p_i \log p_i |_{new} - \sum_{i=k,ne}^{N} p_i \log p_i |_{old} \\
&= \sum_{k=1}^{A} \frac{card(B_{j,k}) - d_u}{card(S)} \log \frac{card(B_{j,k}) - d_u}{card(S)} \\
&+ \sum_{k=1}^{C} \frac{card(B_{j,k}) + d_v}{card(S)} \log \frac{card(B_{j,k}) + d_v}{card(S)} \\
&- \sum_{k=1}^{A} \frac{card(B_{j,k})}{card(S)} \log \frac{card(B_{j,k})}{card(S)} - \sum_{k=1}^{C} \frac{card(B_{j,k})}{card(S)} \log \frac{card(B_{j,k})}{card(S)} \\
&= \sum_{k=1}^{A} \frac{card(B_{j,k}) - d_u}{card(S)} \log \frac{card(B_{j,k}) - d_u}{card(S)} - \frac{card(B_{j,k})}{card(S)} \log \frac{card(B_{j,k})}{card(S)} \\
&+ \sum_{k=1}^{C} \frac{card(B_{j,k}) + d_v}{card(S)} \log \frac{card(B_{j,k}) + d_v}{card(S)} - \frac{card(B_{j,k})}{card(S)} \log \frac{card(B_{j,k})}{card(S)} \\
&= \sum_{k=1}^{A} \left(\frac{card(B_{j,k})}{card(S)} - \frac{d_u}{card(S)}\right) \log \left(\frac{card(B_{j,k})}{card(S)} - \frac{d_u}{card(S)}\right) \\
&- \frac{card(B_{j,k})}{card(S)} \log \frac{card(B_{j,k})}{card(S)} \\
&+ \sum_{k=1}^{C} \left(\frac{card(B_{j,k})}{card(S)} + \frac{d_v}{card(S)}\right) \log \left(\frac{card(B_{j,k})}{card(S)} + \frac{d_v}{card(S)}\right) \\
&- \frac{card(B_{j,k})}{card(S)} \log \frac{card(B_{j,k})}{card(S)}
\end{aligned}
$$

The information entropy change induced by perturbation is given above. We can compare this formula with the total information entropy before the system changes. Because $card(S) = M$, and $d_u, d_v < W \times M \ll M$, it is easy to know that $\frac{d_u}{card(S)}$ and $\frac{d_v}{card(S)}$ are both small quantities.

Therefore, the absolute value of the ratio of the $\Delta\sigma$ and the $\sigma_{old} = -\sum_i^N p_i \log p_i$ will be found:

$$\eta = \left|\frac{\Delta\sigma}{-\sum_i^N p_i \log p_i}\right|$$

$$= \left|\frac{\Delta\sigma}{-\sum_{i\neq k,ne}^N p_i \log p_i |_{old} - \sum_{i=k,ne}^N p_i \log p_i |_{old}}\right| < \left|\frac{\Delta\sigma}{\sum_{i=k,ne}^N p_i \log p_i |_{old}}\right|$$

$$= \left|\frac{\Delta\sigma}{\sum_{k=1}^A \frac{card(B_{j,k})}{card(S)} \log \frac{card(B_{j,k})}{card(S)} + \sum_{k=1}^C \frac{card(B_{j,k})}{card(S)} \log \frac{card(B_{j,k})}{card(S)}}\right|$$

$$= \frac{\Delta\sigma}{\sum_{k=1}^A \frac{card(B_{j,k})}{card(S)} \log \frac{card(B_{j,k})}{card(S)} + \sum_{k=1}^C \frac{card(B_{j,k})}{card(S)} \log \frac{card(B_{j,k})}{card(S)}}$$

It is found that this formula is similar to the differential of $\sigma_{old} = -\sum_i^N p_i \log p_i$. when $d_u, d_v < W \times M \ll M,\ \eta \ll 1$, which indicates that the change of information entropy at this time is a so small amount that negligible. It is worth mentioning that if the magnitude of the perturbation scale L is less than the interval width H_i of the subset B_i, but the probability p_i of the subset B_i is changed, it will also cause similar changes, and such changes are also very small. The analysis process is similar to the above, so it can also be ignored.

3. If the magnitude of the perturbation scale L is much larger than the width H_i of *BIN*, that is $L \gg H$, but in this case the coupling coefficient changes $\Delta\kappa(L)$ caused by the perturbation scale L is not greater than the normalized factor $G = \sum_i \kappa_i$. Such a change is similar to that if the magnitude of the perturbation scale L is close to or equal to the interval width H_i of the subset B_i, the information entropy will change at this time, and such a change is also very small, and the analysis process is similar to the above, so it can also be ignored.

After the above discussion, we can draw a conclusion for the time being in the article:

1. If the magnitude of the coupling coefficient change caused by perturbation scale L is less than the width H of *BIN*, $L \ll H$;

2. If the magnitude of the change in the coupling coefficient caused by the perturbation scale L is close to or equal to the width H of *BIN*, $L \approx H$;

3. If the magnitude of the change in the coupling coefficient caused by the perturbation scale L is much larger than the width H of *BIN*, $L \gg H$, but the coupling coefficient changes $\Delta\kappa(L)$ caused by the perturbation scale L is not greater than the normalized factor G;

In all three cases, the information entropy calculation is robust, and the perturbation hardly changes the entropy, which can reveal why the information entropy remains a constant value in the initial state of the perturbation.

Below we discuss the case if the perturbation scale L is greater than the normalized factor G. In order to explain this point clearly, it is necessary to go back to the normalization step. In the process of using information entropy to calculate the coupling coefficient, the coupling parameter κ_i is not counted, but the coupling parameter after normalization $\frac{\kappa_i}{G}$. For the distribution of the coupling parameter, normalization will not lead to difference between κ_i and $\frac{\kappa_i}{G}$, but the addition of perturbation will have an important impact.

Still considering the above model, the interval width of the *BIN* interval is $H = \frac{1}{N} = 0.1$, we assume that the disturbance suddenly increases, so that the coupling parameters κ_i so that some coupling parameters suddenly become very large. This will cause the

entire normalization factor $G = \sum_i \kappa_i$ to change to $G \rightarrow F \times G$, where F is the ratio of change of coupling coefficient normalization factor. Because the perturbation rate is low, it can be assumed that only a small number of lattice change, while the majority of lattice do not shift. At this point, all the statistical factors have changed dramatically: since the normalized coupling parameters $\frac{\kappa_i}{G}$ are statisticed before the perturbation, and the post-perturbation statistics unit are $\frac{\mathrm{\kappa_i}}{F \times G}$.

After the $G \rightarrow F \times G$ perturbation of the coupling system, the normalized statistical factor is reduced to the original $\frac{1}{F}$ Since the normalized statistical interval is $[0，1]$, and the interval width of each *BIN* interval is $\mathrm{H} = \frac{1}{\mathrm{N}} = 0.1$, after a violent disturbance of $G \rightarrow F \times G$, the majority of lattice factor $\frac{\kappa_i}{F \times G}$ obtained after normalization is less than $\frac{1}{F}$, and all of them fall into the subset B_i interval of $0 - \frac{1}{F}$. At this time, the probability distribution of the system changes greatly, and only some subset $\mathrm{B_i}$ has coupling parameters. If $F = M$, then the information entropy of the system is:

$$\sigma = -\sum_{i}^{2} p_i \log p_i = -\frac{1}{M} \log \frac{1}{M} - \frac{M-1}{M} \log \frac{M-1}{M}$$
$$= \frac{1}{M} \log M + \frac{M-1}{M} \log \left(1 - \frac{1}{M-1}\right)$$

As $\mathrm{M} \rightarrow \infty$, $\sigma \rightarrow 0$. At this time, the information entropy decreases more rapidly. The actual calculation shows that when $\mathrm{F} \geq 2$, the information entropy will suddenly drop.

In fact, for those systems where the size of the coupling coefficient is related to the position, the change of the position will also cause faster changes in the coupling coefficient. For example, the information entropy of a system with an inverse ratio of the eighth power is bound to decay much faster than that of a system with an inverse

ratio of the second power, because in a system with a high inverse ratio, the position perturbation is more likely to cause sudden changes in the system's coupling coefficient. But in general, it can be easily shown that for coupled systems where the coupling coefficient is inversely proportional to the distance, the change in the coupling coefficient due to the proximity of the particles will be much greater than the decrease in the coupling coefficient due to the distance between the particles. We assume that the coupling coefficient κ_i is inversely proportional to the *n* power: $\kappa_i = \kappa_i(r_i) = \lambda {r_i}^{-n}$. For the same positional disturbance L, there will always be:

$$|\kappa_i(r_i + L) - \kappa_i(r_i)| = \lambda {r_i}^{-n} - \lambda(r_i + L)^{-n}$$

$$|\kappa_i(r_i - L) - \kappa_i(r_i)| = \lambda(r_i - L)^{-n} - \lambda {r_i}^{-n}$$

$$|\kappa_i(r_i - L) - \kappa_i(r_i)| > |\kappa_i(r_i + L) - \kappa_i(r_i)|$$

For the random position perturbation process, the total coupling coefficient of the system will always increase, and this will be especially aggravated with the increase of the perturbation scale, eventually lead to broken of the robustness, which brings us to the fourth conclusion:

4. According to this example, if the perturbation scale L is greater than the normalization factor *G*, the information entropy will decrease at a very fast rate, which also marks the broken of the robustness of the information entropy statistics.

In summary, the information entropy can be used to describe the robustness of topological systems.

III.The extension of information entropy method in other topological systems,

including Su-Schrieffer-Heeger (SSH) model and the valley-Hall photonic crystal.

The Kagome model has been analyzed with information entropy in the article, and this method can be extended to other systems, such as SSH model and the valley-Hall photonic crystal. The result of SSH model and the valley-Hall photonic crystal will provide here. The information entropy method can be extended to other topological systems.

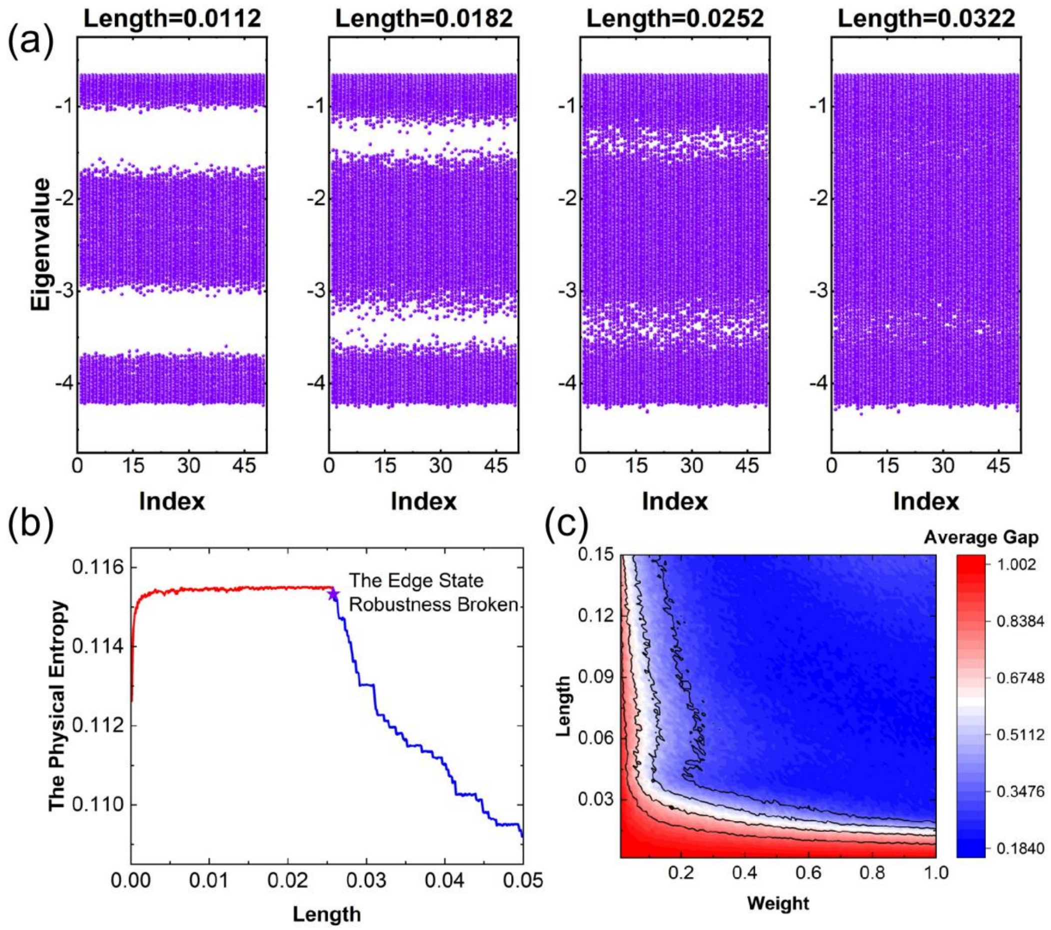

Figure SM1. (a)The band gap state of the SSH model with different perturbation scale Length. (b)The variation of physical entropy with Length of the ~~two~~ model, and the point where the robustness is broken corresponds to the point where the information entropy decreases swiftly. (c) The diagram of the phase transition of the SSH model. The red area represents the topological phase and the blue area represents the trivial phase.

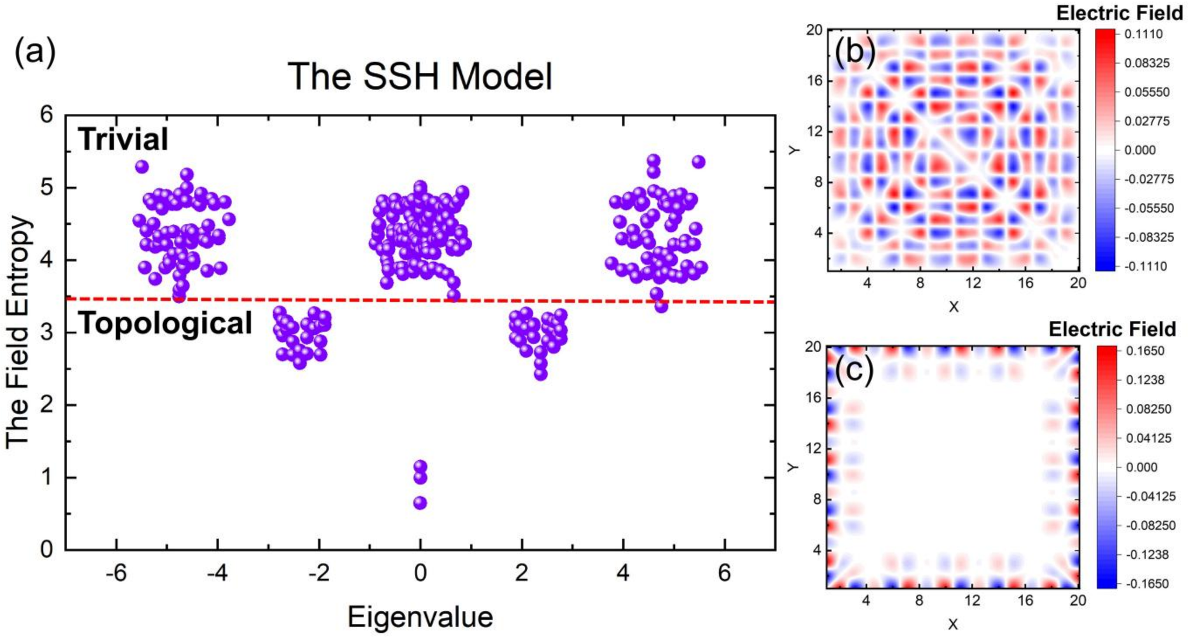

Figure SM2. (a)The electric field entropy of the SSH model. The electric field distribution of the topological states are the states with a low electric field entropy, and the bulk states is a series of states with a high entropy of electric field entropy. (b) The trivial electric field of the SSH model; (c)The topological electric field of the SSH model.

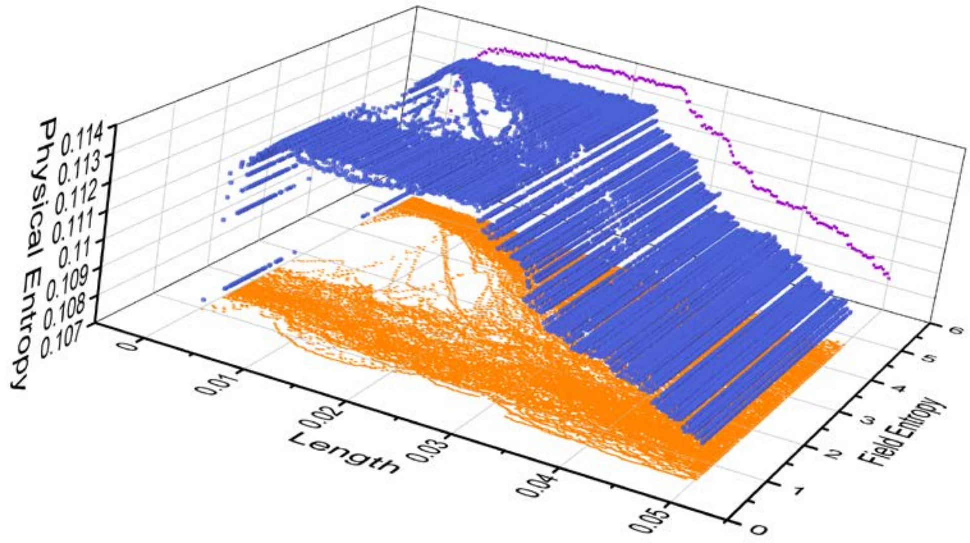

Figure SM3. The relationship between the physics entropy and the electric field entropy of the SSH model. The blue points are the relationship between the physics entropy and the field entropy. The origin points are the projector of the blue points, meaning the field entropy of the SSH model with the perturbation scale growing. The process of the electric field entropy gap closing can be observed. And the purple points are variation of physical entropy with *Length* of the SSH model.

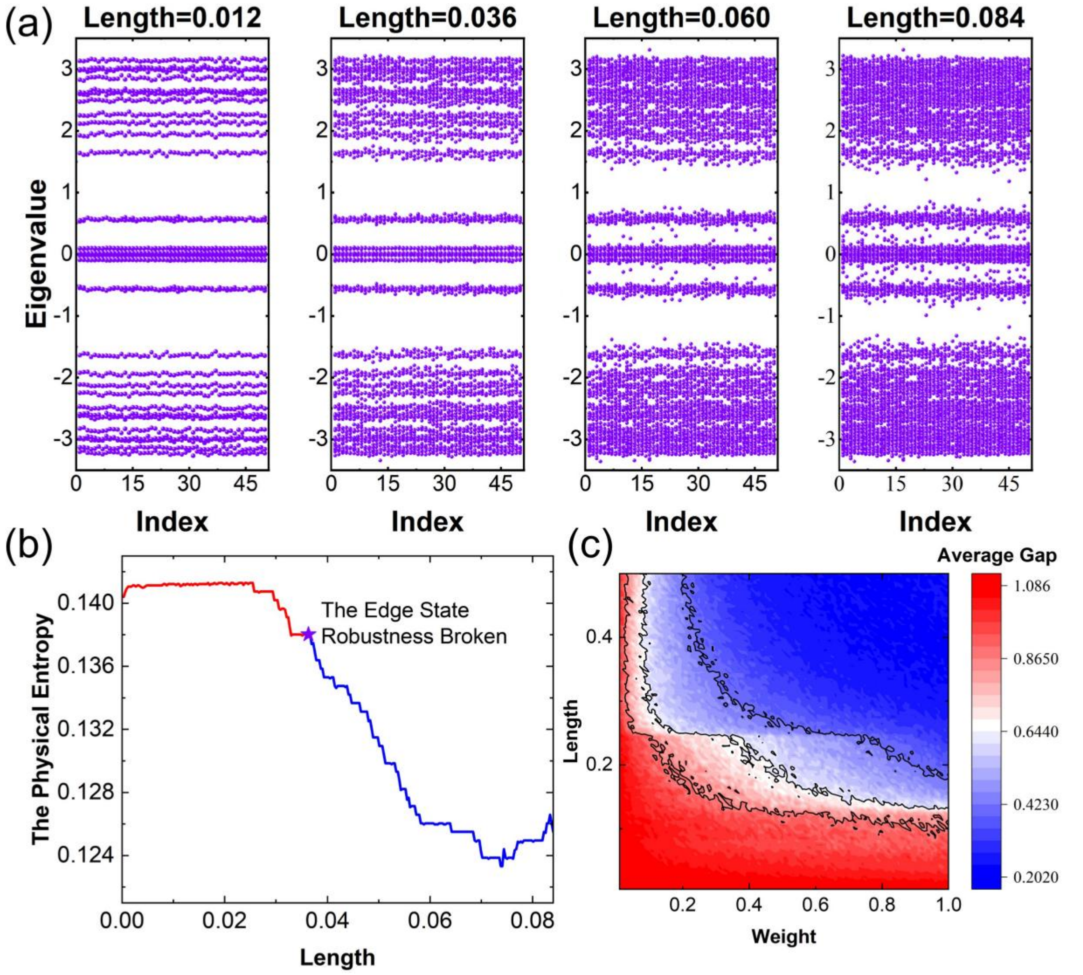

Figure SM4. (a)The band gap state of the valley-Hall photonic crystal with different perturbation scale Length. (b)The variation of physical entropy with Length of the ~~two~~ model, and the point where the robustness is broken corresponds to the point where the information entropy decreases swiftly. (c) The diagram of the phase transition of the valley-Hall photonic crystal. The red area represents the topological phase and the blue area represents the trivial case.

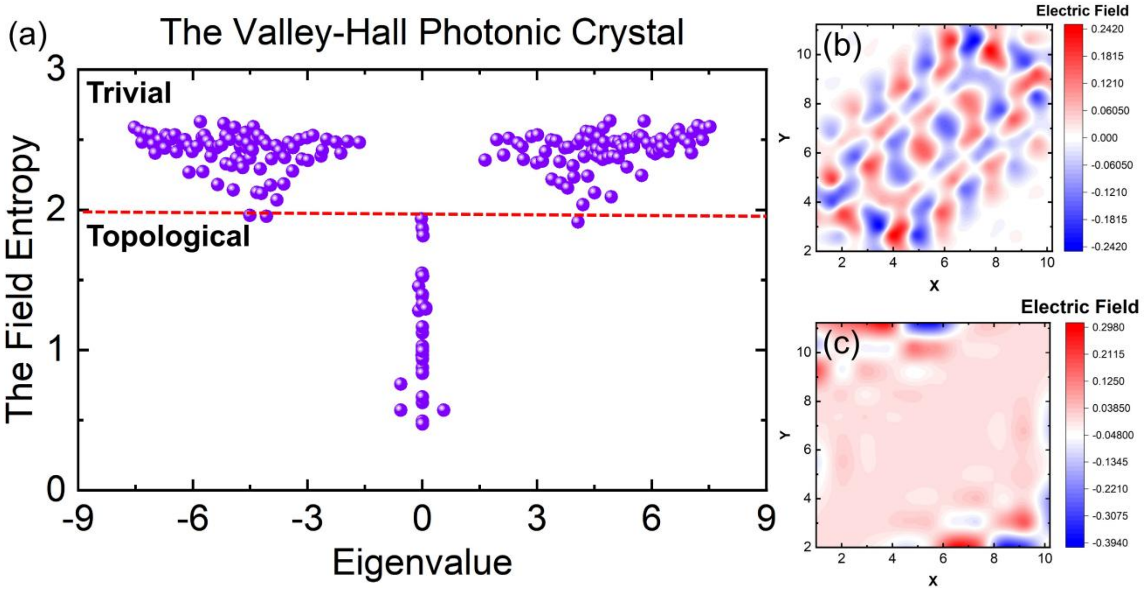

Figure SM5. (a)The electric field entropy of the valley-Hall photonic crystal. The electric field distribution of the topological states are the states with a low electric field entropy, and the bulk states is a series of states with a high entropy of electric field entropy. (b) The trivial electric field of the valley-Hall photonic crystal; (c)The topological electric field of the valley-Hall photonic crystal.

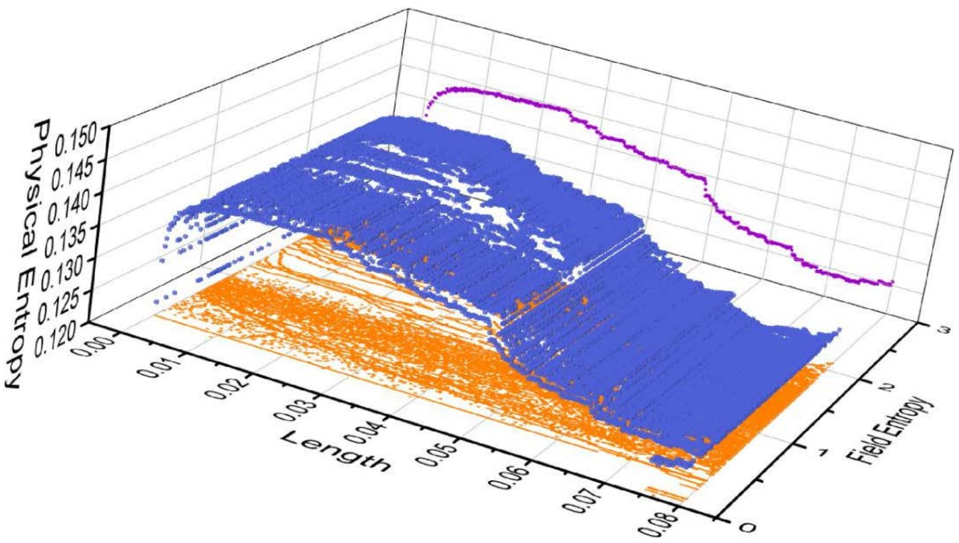

Figure SM6. The relationship between the physics entropy and the electric field entropy of the valley-Hall photonic crystal. The blue points are the relationship between the physics entropy and the field entropy. The origin points are the projector of the blue points, meaning the field entropy of the valley-Hall photonic crystal with the perturbation scale growing. The process of the electric field entropy gap closing can be observed. And the purple points are variation of physical entropy with *Length* of the valley-Hall photonic crystal.

IV. The discussion about the information entropy method can be generalized to other complex lattice models and the potential of lattice site in coupling system.

With the previous discussion, the information entropy method can be applied in the three common simple lattices (Kagome, SSH and valley hall model). Further, the information entropy method is also can be generalized to other complex lattice models, and a discussion about the case that the disturbance is applied on the potential of lattice site in the Kagome model is also worth provided. Here is the result about the information entropy method can be generalized to other complex lattice models and the potential of lattice site in coupling system.

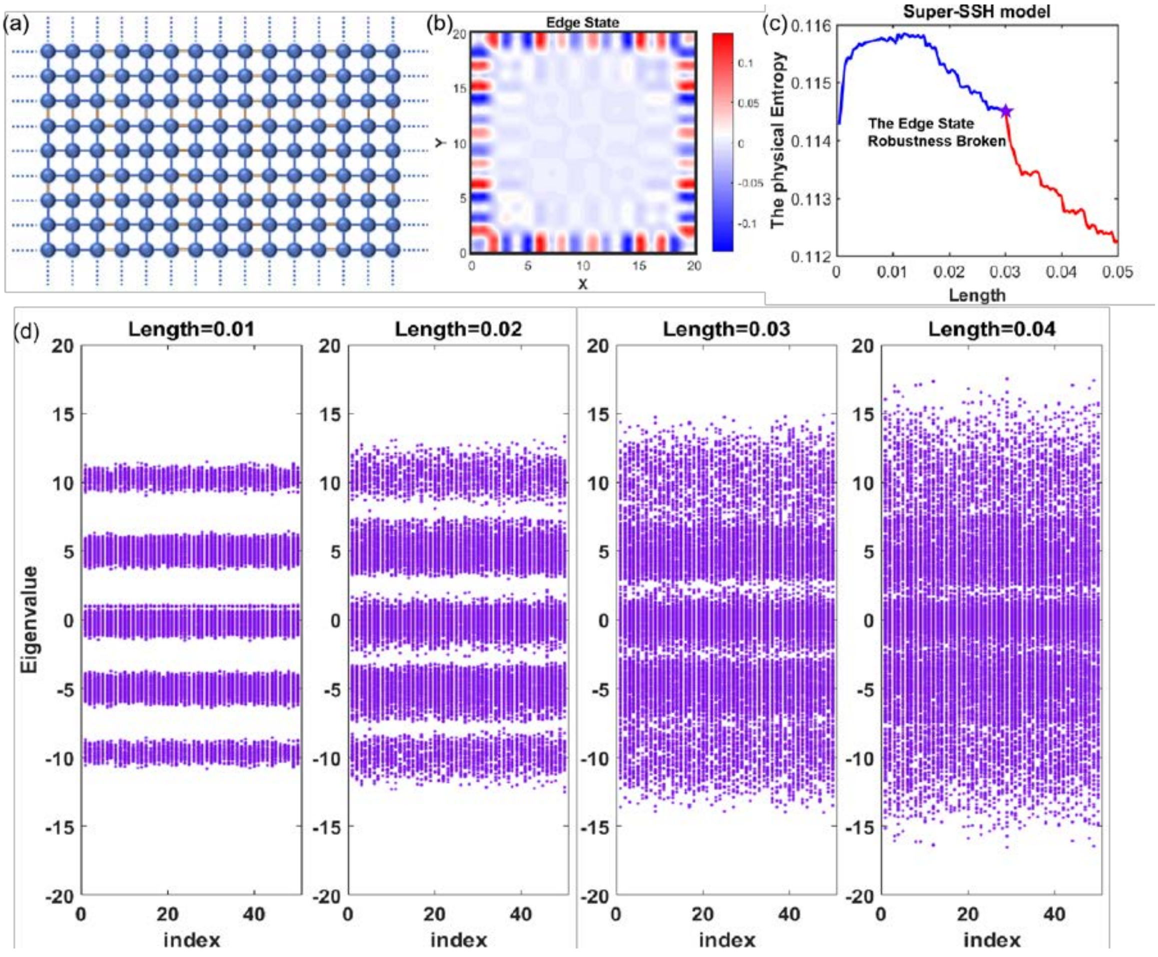

Figure SM7. (a)The two-dimensional super-SSH model under periodic boundary condition[29]. (b)The topological electric field of the super-SSH model. (c) The variation of PE with Length of

the super-SSH model, and the point where the edge state disappearing correspond to the point where IE decreases swiftly. (d)The band gap state of the super-SSH model with different perturbation scale Length.

Conceptually, for a complex lattice, some different coupling coefficients can be treated as an equivalent-effective coupling coefficient as a whole, and then the complex lattices can be analyzed as the common simple lattices with the information entropy method. Therefore, the information entropy method is also suitable for more complex periodic complex lattice. The information entropy theory mentioned in Supplement 2 is also applicable in complex lattices.

For the case of complex lattice, some relevant calculations are carried out. Because the difference between complex lattice and the three common lattices mentioned in the paper (Kagome, SSH and valley hall model) is mainly reflected in the change of coupling coefficient rather than the local characteristics of the electric field distribution of the topological edge state. Therefore, the method of electric field entropy is still applicable to the topological electric field of complex lattice. The application of the physical entropy in the complex lattices will be mainly discussed.

For convenience, the Super-SSH model is selected to carry out the calculation. The coupling structure is shown in Figure SM7(a). Figure SM7(b) shows electric field distribution of the topological edge state of Super-SSH. The Super-SSH model can be seen that for a compound lattice like of SSH. After reasonably dividing the Bin and discreting the system coupling coefficients, the physical entropy of the band-gap closure position in the statistical sense will still show a rapid decline, and the initial state is still the topological state of the system, as shown in Figure SM7(c). This shows

that the information entropy method is still applicable for periodic complex lattices.

When a system has the potential of lattice site in coupling system, the information entropy method is also applicable in principle. For the Hamiltonian of a coupled system, the coupling coefficient mainly affects the non-diagonal terms of the Hamiltonian matrix, while the potential of lattice site mainly affects the diagonal terms of the Hamiltonian matrix. The physical entropy method proposed is to carry out statistics on the matrix elements in the Hamiltonian matrix, without being confined to the diagonal and non-diagonal terms in the Hamiltonian matrix. Therefore, for the physical system considering the potential of lattice site, the physical entropy method can also perform statistics on the system.

However, the application of information entropy method is conditional. The potential of lattice sites is randomly applied to kagome lattice for calculation. The statistical behavior of the band gap and physical entropy behavior of the system can be studied by changing the size of the potential of lattice sites. The calculation results show that if the lattice potential in the kagome lattice has a similar or smaller order of magnitude compared with the coupling coefficients in the system, the band gap of the system will not be greatly affected. The calculation results are shown in Figure SM8(a) and (b), and the diagonal term is added to the Hamiltonian at this time. Because the potential of lattice site is small and the coupling coefficient has a great influence on the band gap, the physical entropy still has a good characteristic of the topological phase transition, which will still decrease rapidly at the position where the band gap is closed on the statistical sight. However, when the lattice potential magnitude far exceeds the coupling

coefficient magnitude in the system, then the lattice potential will directly affect the band gap of the system, and it will occupy a crucial position, while the confusion of the coupling coefficient caused by perturbation will be in a secondary position. At this time, the calculation results are shown in Figure SM8(c) and (d). Since the information entropy method mainly deals with the confusion of the system coupling coefficient, when the lattice potential is dominant, it is difficult for the information entropy method to correspond to the bandgap behavior.

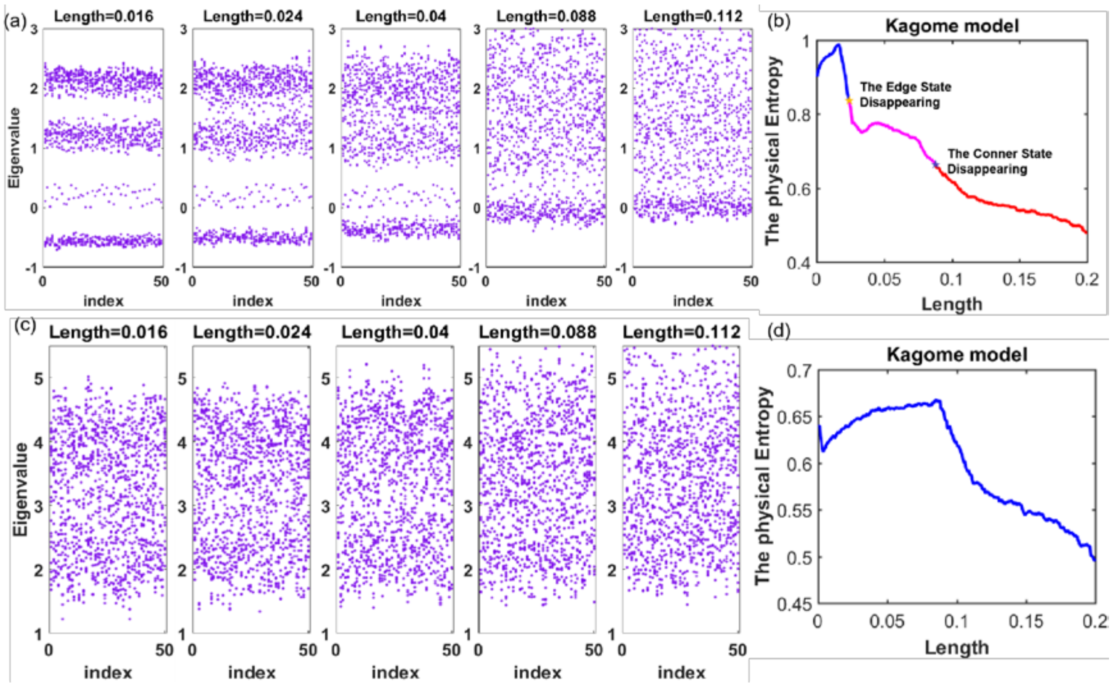

Figure SM8. (a)The band gap state of the Kagome model with different perturbation scale Length when the lattice potential in the kagome lattice has a similar or smaller order of magnitude compared with the coupling coefficients in the system. (b)The variation of PE with Length of the Kagome model when the lattice potential in the kagome lattice has a similar or smaller order of magnitude compared with the coupling coefficients in the system. The physical entropy still has a good characteristic of the topological phase transition. (c) The band gap state of the Kagome model with different perturbation scale Length when the lattice potential magnitude far exceeds the coupling coefficient magnitude in the system. (d)The variation of PE with Length of the Kagome model when the lattice potential magnitude far exceeds the coupling coefficient magnitude in the system. It is difficult for the information entropy method to correspond to the band gap behavior at that time.

In conclusion, the extension and limitations of the information entropy method can be discussed. Since the current calculation is carried out for the periodic simple lattice and complex lattice in the coupling system, it can be considered that the current information entropy method is suitable for the simple lattice and complex lattice in the periodic system. Since the concept of entropy is derived from statistical mechanics, it has potential advantages in describing huge disordered amorphous systems. However, according to the information entropy theory, for the system with extremely strong coupling coefficients or extremely strong lattice potential, the confusion of the system coupling coefficients will no longer be the main reason affecting the band gap of the system. Therefore, in this case, the information entropy method has certain limitations.
